# Frequency of contact with confidants and cognitive decline over time in old age

**DOI:** 10.1093/geroni/igaf122.3110

**Published:** 2025-12-31

**Authors:** Veronica Howell, Benjamin Shaw, Uchechi Mitchell

**Affiliations:** University of Illinois Chicago, Chicago, Illinois, United States; University of Illinois Chicago, Chicago, Illinois, United States; University of Illinois Chicago, Chicago, Illinois, United States

## Abstract

Network size and frequency of contact are both positively associated with cognitive function among older adults, however intervention recommendations stemming from these associations remain relatively vague. For example, preventing social isolation is a common goal for reducing dementia risk, but our current conceptualization of the personal network characteristics that constitute social isolation remains imprecise. The purpose of this study is to add precision to our understanding of links between personal network factors and cognitive decline, thus highlighting potential targets for preventing social isolation. Data are from the National Social Life, Health, and Aging Project. Round 1 (2005) network size and frequency of contact measures were combined to create a new set of measures describing: number of confidants with whom the respondent talks frequently (daily or multiple times per week), periodically (weekly or monthly), and seldomly (< monthly). Associations between these measures and cognitive function 5 and 10 years later were estimated while controlling for baseline cognitive function and other covariates. On average, respondents identified 2.5 frequently contacted confidants, 1.5 periodically contacted confidants, and 1 seldomly contacted confidant. Number of periodically contacted confidants was most strongly associated with future cognitive function (p<.001). These findings suggest that maintaining contact with confidants outside of one’s daily circle may be an important way to prevent social isolation and its associated dementia risk. More research is needed to understand why periodically contacted social ties might bring cognitive benefits, with likely explanations involving regular exposure to diverse information and perspectives while limiting opportunities for interpersonal stress.

